# ICG-augmented hyperspectral imaging for visualization of intestinal perfusion compared to conventional ICG fluorescence imaging: an experimental study

**DOI:** 10.1097/JS9.0000000000000706

**Published:** 2023-09-14

**Authors:** Alexander Studier-Fischer, Florian Marc Schwab, Maike Rees, Silvia Seidlitz, Jan Sellner, Berkin Özdemir, Leonardo Ayala, Jan Odenthal, Samuel Knoedler, Karl-Friedrich Kowalewski, Caelan Max Haney, Maximilian Dietrich, Gabriel Alexander Salg, Hannes Götz Kenngott, Beat Peter Müller-Stich, Lena Maier-Hein, Felix Nickel

**Affiliations:** Departments ofa General, Visceral, and Transplantation Surgery; b Anesthesiology, Heidelberg University Hospital; c Division of Intelligent Medical Systems, German Cancer Research Center (DKFZ); d Faculty of Mathematics and Computer Science; e Medical Faculty, Heidelberg University; f HIDSS4Health—Helmholtz Information and Data Science School for Health, Karlsruhe; g National Center for Tumor Diseases (NCT) Heidelberg, a partnership between DKFZ and Heidelberg University Hospital, Heidelberg; h Department of Urology, Medical Faculty of Mannheim at the University of Heidelberg, Mannheim; i Department of General, Visceral, and Thoracic Surgery, University Hospital Hamburg-Eppendorf, Hamburg, Germany; j Division of Plastic Surgery, Department of Surgery, Brigham and Women’s Hospital, Harvard Medical School, Boston, MA

**Keywords:** HSI, hyperspectral imaging, ICG, indocyanine green, perfusion study, pig, small bowel ischaemia, translational research

## Abstract

**Background:**

Small bowel malperfusion (SBM) can cause high morbidity and severe surgical consequences. However, there is no standardized objective measuring tool for the quantification of SBM. Indocyanine green (ICG) imaging can be used for visualization, but lacks standardization and objectivity. Hyperspectral imaging (HSI) as a newly emerging technology in medicine might present advantages over conventional ICG fluorescence or in combination with it.

**Methods:**

HSI baseline data from physiological small bowel, avascular small bowel and small bowel after intravenous application of ICG was recorded in a total number of 54 in-vivo pig models. Visualizations of avascular small bowel after mesotomy were compared between HSI only (1), ICG-augmented HSI (IA-HSI) (2), clinical evaluation through the eyes of the surgeon (3) and conventional ICG imaging (4). The primary research focus was the localization of resection borders as suggested by each of the four methods. Distances between these borders were measured and histological samples were obtained from the regions in between in order to quantify necrotic changes 6 h after mesotomy for every region.

**Results:**

StO_2_ images (1) were capable of visualizing areas of physiological perfusion and areas of clearly impaired perfusion. However, exact borders where physiological perfusion started to decrease could not be clearly identified. Instead, IA-HSI (2) suggested a sharp-resection line where StO_2_ values started to decrease. Clinical evaluation (3) suggested a resection line 23 mm (±7 mm) and conventional ICG imaging (4) even suggested a resection line 53 mm (±13 mm) closer towards the malperfused region. Histopathological evaluation of the region that was sufficiently perfused only according to conventional ICG (R3) already revealed a significant increase in pre-necrotic changes in 27% (±9%) of surface area. Therefore, conventional ICG seems less sensitive than IA-HSI with regards to detection of insufficient tissue perfusion.

**Conclusions:**

In this experimental animal study, IA-HSI (2) was superior for the visualization of segmental SBM compared to conventional HSI imaging (1), clinical evaluation (3) or conventional ICG imaging (4) regarding histopathological safety. ICG application caused visual artifacts in the StO_2_ values of the HSI camera as values significantly increase. This is caused by optical properties of systemic ICG and does not resemble a true increase in oxygenation levels. However, this empirical finding can be used to visualize segmental SBM utilizing ICG as contrast agent in an approach for IA-HSI. Clinical applicability and relevance will have to be explored in clinical trials.

**Level of evidence:**

Not applicable. Translational animal science. Original article.

## Introduction

Highlights

- Small bowel malperfusion can be identified with hyperspectral imaging (HSI).
- Indocyanine green (ICG) can be used as contrast agent with HSI.
- Evaluation with ICG-augmented HSI is even more sensitive for detecting malperfusion.
- ICG-augmented HSI can increase patient safety for malperfused small bowel resection.

Small bowel malperfusion (SBM) causes high morbidity and has severe surgical consequences^1^. This can result in a reduced quality of life, significant morbidity and high rates of readmission as well as reoperation^2^. Regarding the pathomechanism, there are occlusive (arterial embolism, arterial or venous thrombosis) as well as non-occlusive forms of SBM, of which arterial origins are far more common than venous^3^. Regarding localization there are three principal scenarios that can be differentiated. Malperfusion can either originate through local injury to the mesentery^1^ (e.g. septic emboli, stabbing wound), resulting in rather absolute and segmental malperfusion. Alternatively, it can originate through processes at the root of the mesentery^2^ (e.g. tumour, macrovascular atherosclerosis) or lastly by systemic disease^3^ (e.g. cardiocirculatory insufficiency, septic shock or high catecholamines dosage) resulting in a functional and more generalized malperfusion with a potentially worse outcome^3,4^.

Small bowel has great compensation capacities regarding the amount of blood flow required in order to keep physiological metabolism and structural wall integrity. Its blood flow has to experience a 75% reduction from its physiological level over several hours for irreversible intestinal ischaemia to occur^4^. This trait might have been acquired over the course of evolution due to the periodically greatly fluctuating workload depending on its primary function that is digestion. This resistance to malperfusion brings a great advantage for small bowel; however, it also causes a relevant diagnostic gap at the same time in that patients can be asymptomatic during initial stages of malperfusion and only become clinically apparent when tissue damage is already severe with triggering events long in the past.

Due to its lack of specific early onset symptoms and laboratory results, the establishment of an early diagnosis is often rendered difficult, while late radiological signs for example intestinal pneumatosis, missing contrast agent or perforation are clear and obvious and trigger immediate surgery^3^.

Especially in case of segmental SBM, recovery can be achieved by strict resection of the affected section. However, there is no objective measuring tool for determining the exact resection borders required for sufficient healing. Thus, surgeons might extend the resection margins to areas of safe perfusion at the cost of bowel length and intestinal resorption capacity. In case of multiple malperfused bowel segments, resection should be restricted to avoid short-bowel syndrome.

Visualization of SBM and intraoperative decision-making for resection margins is not trivial. While contrast-enhanced computer tomography is well-suited to categorically diagnose SBM previous to surgery, intraoperative evaluation of the extent is usually done by clinical inspection through the surgeon focusing on colour and peristalsis. There are advanced imaging technologies such as laser speckle^5,6^ or indocyanine green (ICG) imaging^5,7–16^ that have been extensively investigated for this purpose. Yet, they have sparsely found their way into clinical routine.

Especially ICG seems to have diagnostic advantages in that it can provide definite confirmation of SBM through absence of ICG fluorescence in malperfused regions. It was originally developed as a dye in photography in the Second World War and first tested for clinical use at the Mayo Clinic in 1957. It is known to be a nontoxic, almost non-allergenic^17,18^ and unstable compound bound by albumin in the intravascular space and has a biological half-life of about 3–4 min. Initial application was in diagnostics of hepatic function and cardiology^19^, while in the meantime it has become established as the standard for example fluorescent angiography in ophthalmology. Since then, it has adopted multiple roles in medicine, not only as a chromophore and contrast agent, but also as a photosensitizer for therapeutic use^20^.

Existing scientific evidence in the form of case reports as well as retrospective studies, makes it very clear that the absence of ICG in small bowel equals absence of perfusion and will ultimately result in bowel necrosis, if there is no possibility for intervention and revascularization^7,11–13,16^. However, a retrospective analysis of 52 patients also revealed two cases of false negative evaluation and although ICG appeared in the bowel segments, these ultimately perished^11^. This is a common phenomenon that is experienced regularly, but not reported or published. Even more, other studies underline that different ICG systems can also show heterogenous results^21^. It is therefore known that ICG analyses are heterogenous and can overestimate perfusion, which is why there are studies trying to optimize ICG analyses using advanced methods such as time-to-peak analyses without translation into clinical routine^22^. Hence, for these reasons a diagnostic gap can be assumed with cases of overestimation of perfusion when using ICG.

Hyperspectral imaging (HSI) as a newly emerging technology in medicine uses reflectance intensities over a broad range in the electromagnetic spectrum. It does not rely on external contrast agents, such as ICG, for the visualization of tissue oxygenation, but it is nevertheless able to visualize these contrast agents additionally. In its basic application without ICG, HSI captures spectral fingerprints of tissue pigments and chromophores such as haemoglobin. In the past, these spectral features were shown to be highly characteristic for organ entities^23–25^. However, as the physiology of ICG is so unbribably intertwined with tissue perfusion, it bears the question whether ICG-augmented HSI (IA-HSI) offers diagnostic value. IA-HSI combines benefits from organ-unspecific spectral changes caused by ICG with benefits from organ-specific spectral changes caused by malperfusion and might present significant advantages over conventional ICG imaging. Therefore, IA-HSI has the potential of closing the aforementioned diagnostic gap (Supplement Figure 1, Supplemental Digital Content 1, http://links.lww.com/JS9/A972)^26–29^. Here, we hypothesize that IA-HSI is more sensitive for the visualization of SBM extent compared to conventional ICG imaging.

## Materials and methods

### Animals and anaesthesia

This animal study was approved by the Committee on Animal Experimentation of the regional council. All experimental animals were managed according to the directives of the European Community Council (2010/63/EU), ARRIVE guidelines^30^ and according to German laws for animal use and care.

Data of a total maximum number of 64 animals were included in the analysis as indicated in the figures. Male pigs (“Sus scrofa ssp. Domesticus”) with a mean weight of 35.2 kg were used as experimental animals. They were acclimatized for 48 h in an enriched environment following circadian cycles of light and dark periods, had free access to water and were fed regularly until being starved 24 h prior to surgery. Pharmaceutical treatment and narcosis (but also other protocols such as for corrosion casting and histopathology) were performed according to institution standard as described in earlier publications^23,25^. Body weight adapted pharmacological calculations are generalized for a 40 kg pig. Initial sedation was performed with a weight adapted intramuscular injection of the neuroleptic azaperone (Stresnil 40 mg/ml by Elanco) with 6 mg/kg (≈ 6 ml = 240 mg) 15 min prior to further manipulation. Next, analgosedation was established by a weight adapted intramuscular injection of a combination of both the short-acting benzodiazepine midazolam (Midazolam-hameln 5 mg/ml by hameln pharma plus gmbh) with 0,75 mg/kg (≈ 6 ml = 30 mg) and ketamine (Ketamin 10% by Heinrich Fromme) with 10 mg/kg (≈ 4 ml = 400 mg).

Intubation was performed conventionally or via tracheotomy in case of reduced laryngeal visibility. Medication used during intubation in case of excessive sputum production or general backup medication included intravenous (i.v.) atropine and propofol 1%. After intubation pressure-controlled ventilation was established and a minimal alveolar concentration of 1.1 was achieved under sevoflurane. Intraoperative anaesthesia was achieved through balanced narcosis with sevoflurane and the combination of i.v. 0.2 mg/kg/h midazolam (≈ 1.5 ml/h = 7.5 mg/h) and 8.75 mg/kg/h ketamine (≈ 3.5 ml/h = 350 mg/h) at a rate of 5 ml/h. No relaxant agents were applied.

Monitoring included pulse oximetry, capnometry and invasive blood pressure via the femoral artery in order to prevent measuring false data resulting from impaired circulation. Mean arterial pressure in the aorta at the time of measurements was 71 mmHg (±6 mmHg). The lowest mean arterial pressure measured during recordings was 62 mmHg. Body temperature was monitored and maintained with electrically controlled heat blankets.

After surgery pigs were euthanized with a rapid i.v. application of 50 ml of potassium chloride solution. Death was pronounced upon an end-expiratory CO_2_ partial pressure below 8 mmHg. All animals and measured datapoints were used in the analyses. There were no different experimental groups. Due to the highly comparable small bowel physiology to humans, results are expected to generalize correctly.

### Surgical procedure

**Figure 1:**
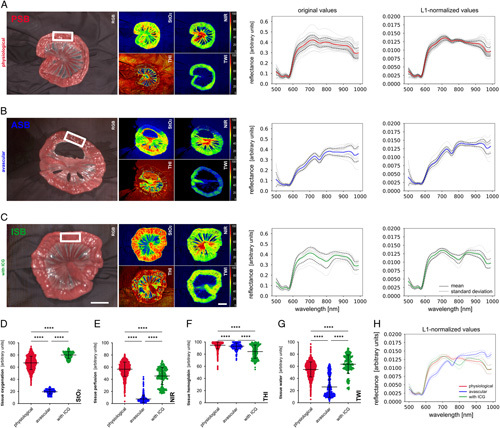
Small bowel baseline data. oxygenation index (StO_2_), near-infrared perfusion index (NIR), tissue haemoglobin index (THI) the tissue water index (TWI) recordings with original and L1-normalized reflectance. (A) Physiological small bowel (PSB) (A=54, *n*=1595). (B) Avascular small bowel (ASB) (A=17, *n*=531). (C) Small bowel after intravascular (i.v.) application of ICG (ISB) (A=13, *n*=256). The white rectangle shows a representative region for the measurements. (D) Quantification of StO_2_. (E) Quantification of NIR. (F) quantification of THI. (G) Quantification of TWI. (H) comparison of median spectra with standard deviation. Boxplots show mean and standard deviation. A indicates number of animals, n indicates number of independent measurements. Scale bar equals 5 cm. ICG, indocyanine green.

Midline laparotomy was performed to access the abdominal cavity. For the baseline recordings, small bowel loops were manually exposed to create physiological recordings. Segmental mesotomy was performed with a bipolar vessel-sealing device (LigaSure) to create baseline avascular small bowel recordings. ICG application was performed as described below and recordings were made between 3 and 15 min after application to create baseline small bowel recordings with ICG (Fig. 1).

**Figure 2:**
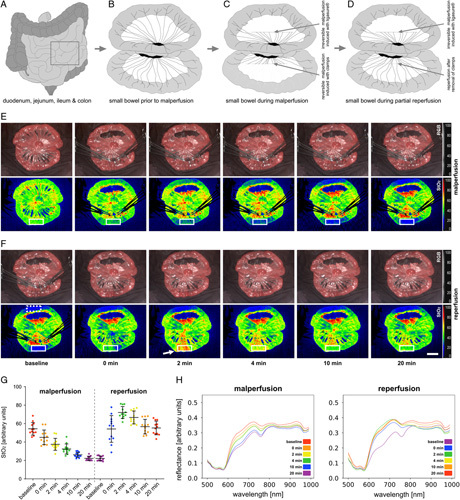
Small bowel malperfusion and reperfusion. (A) Schematic overview of intestinal organs. (B) Depiction of two small bowel loops prior to segmental impairment of perfusion. (C) Depiction of two small bowel loops with irreversible mesotomy-induced (cranial) and reversible clamp-induced (caudal) segmental impairment of perfusion. (D) Depiction of two small bowel loops with irreversible segmental impairment of perfusion (cranial) and reperfusion (caudal). (E) StO_2_ images of segmental impairment of perfusion. (F) StO_2_ images of reperfusion. White arrow shows reactive hyperaemia. (G) quantification of StO_2_ in the region highlighted with the white rectangle in (E) and (F) (A=12). The region highlighted with the dashed white rectangle served as a reference region. (H) Corresponding spectral reflectance. Boxplots show mean and standard deviation. A indicates number of animals. Scale bar equals 5 cm.

Next, two loops were placed adjacent to one another. The upper bowel loop served as the reference loop and received irreversible mesotomy-induced segmental impairment of small bowel perfusion, while the lower bowel loop received reversible clamp-induced segmental impairment of small bowel perfusion (Fig. 2). Both were recorded over a duration of 20 min (“malperfusion”) and for another 20 min after release of the clamp on the lower small bowel loop (“reperfusion”). Reperfusion baseline was recorded after 21 min into malperfusion.

**Figure 3:**
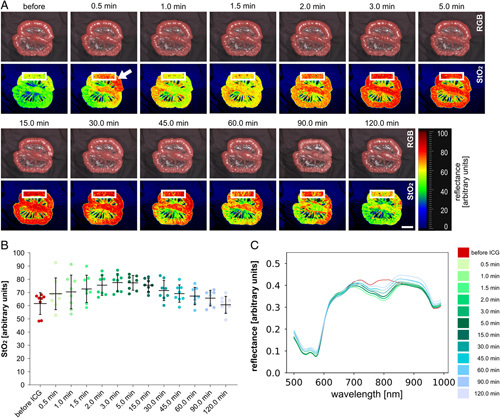
ICG progression over time. (A) Visualization of ICG effects on small bowel loop over time. (B) Quantification of StO_2_ values in the region highlighted with the white rectangle in a (A=8). (C) Changes of mean spectral reflectance over time after ICG application (A=8). Boxplots show mean and standard deviation. A indicates number of animals. Scale bar equals 5 cm. ICG, indocyanine green.

Now, two new loops were placed adjacent to one another and i.v. ICG was applied as described below. Recordings were done before as well as 0.5, 1.0, 1.5, 2, 3, 5, 15, 30, 45, 60, 90 and 120 min after application (Fig. 3). Changes of small bowel spectral reflectance over time were recorded during 20 min of clamp-induced impaired perfusion and reperfusion as well as over a duration of 120 min after the application of ICG.

**Figure 4:**
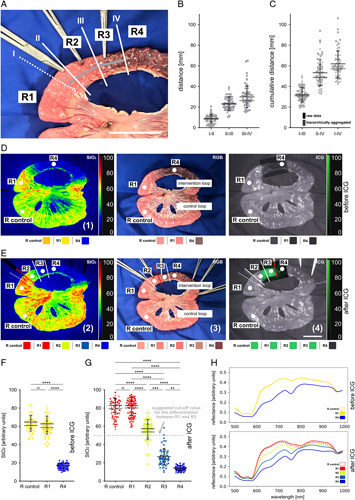
Evaluation of mesotomy-induced segmental impairment of small bowel perfusion. Evaluation before and after ICG application using hyperspectral imaging and conventional ICG optics (A=10, *n*=68). (A) RGB image with indicated borders that could be identified using different imaging modalities. (B) Quantification of distance between borders. (C) Quantification of cumulative distance between borders. (D) Visualization before ICG application. (E) Visualization after ICG application. (F) Quantification of StO_2_ before ICG application. (G), quantification of StO_2_ after ICG application. Grey dashed line indicates the StO_2_ cutoff value that was retrospectively fitted for this dataset to differentiate between R1 and R3 most effectively. (H) Changes in spectral reflectance. Boxplots show mean and standard deviation for raw data (black) and hierarchically aggregated data (grey). Individual pigs are shape coded. A indicates number of animals, *n* indicates number of independent measurements. Scale bar equals 5 cm. ICG, indocyanine green.

As a final experiment, irreversible mesotomy-induced segmental impairment of small bowel perfusion was created in 10 animals. Conventional HSI recordings were performed (1). Now ICG was applied as described below and after 30 s simultaneous recordings with HSI (now IA-HSI) (2) and clinical evaluation (3) as well as conventional ICG imaging (4) were performed (Fig. 4). Borders were now defined as following: (I) height of last perpendicular capillary inflow, (II) height of IA-HSI indicating same StO_2_ levels as compared to healthy reference tissue, (III) height of clinical evaluation through a surgeon where to perform resection and (IV) height of ICG imaging suggesting resection by indicating definitive presence of ICG. Direct as well as cumulative distances between these borders were now quantified.

### HSI system and annotations

The hyperspectral datacubes were acquired by the TIVITA Tissue system from Diaspective Vision GmbH (Am Salzhaff), which is a CMOS push-broom scanning imaging system. It was the first commercially available HSI camera for medicine and provides a high spectral resolution of 5 nm in the visible as well as near-infrared range from 500 to 1000 nm resulting in 100 spectral values along the third dimension (*λ*). Its field-of-view contains 480 × 640 pixels with a spatial resolution of ~0.45 mm/pixel (Supplement Figure 1, Supplemental Digital Content 1, http://links.lww.com/JS9/A972). The distance of the camera to the specimen is controlled via a red-and-green light targeting system. Six halogen spots directly integrated into the camera system provide a standardized illumination. Recording takes around seven seconds plus preheating. During this time external light sources need to be switched off to eliminate interference. Numerical values that are stored in the datacube represent the reflectance values of every single pixel at every spectral measuring step consisting of a total number of 30 720 000 values typically reaching from just above 0 to a maximum of 0.7. These values can be approximately transformed to absorbance by using the negative decadic logarithm resulting in values mostly between above 0 and 4.0. The four original colour-coded index images include the oxygenation index (StO_2_), the near-infrared perfusion index (NIR), the tissue haemoglobin index (THI) and the tissue water index (TWI) and their underlying formulas and calculation can be reviewed in cited literature^31^. Values provided in the text were obtained after hierarchical aggregation across all pigs. A schematic visualization of optical properties of light sources, chromophores and ICG can be seen in the supplement (Supplement Figure 4, Supplemental Digital Content 1, http://links.lww.com/JS9/A972). Annotations were performed by a medical expert. Inter-rater comparison with annotations from a second medical expert revealed only marginal differences (Supplement Figure 5, Supplemental Digital Content 1, http://links.lww.com/JS9/A972).

### Fluorescence imaging system

The fluorescence imaging system used for this experimental study consisted of a light source (D‐Light P Laserfree Lightsource (201337 20)), a camera control unit (IMAGE 1 CONNECT TC 200 + IMAGE 1 H3-LINK TC 300), a camera head (IMAGE1 HD H3-Z FI TH102), ICG optics (HOPKINS Forward-Oblique Telescope 10 mm 30° (26003 BGA)) and a light cable (Fiber Optic Light Cable (495NCSC)) all by Karl Storz (Tuttlingen, Germany). ICG is an anionic tricarbocyanine molecule with fluorescent properties when exposed to excitation lights between 750 and 800 nm. It immediately binds with serum albumin, the most abundant protein in blood and has a relatively short half-life into the bloodstream (3–5 min). When bound, it emits light between 800 and 840 nm after excitation^32–34^. VERDYE (5 mg/ml) from Diagnostic Green GmbH was the ICG used for these experiments. It was dissolved in distilled water prior to application. Several recordings from different angles were made while the room was as dark as feasible. For ICG recordings an i.v. amount of 0.3 mg/kg (≈ 12 mg) ICG was applied.

### Histopathological analysis

**Figure 5:**
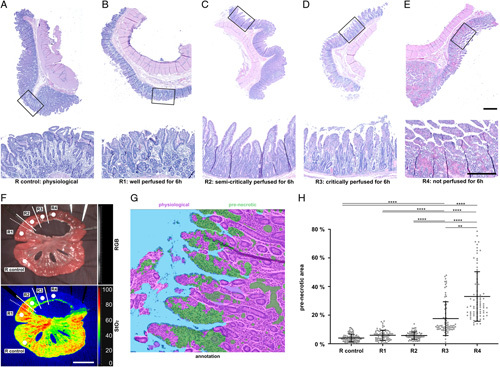
Histopathological correlation of segmental small bowel malperfusion. Histopathological samples from different small bowel regions (A=3). (A) R control: physiological. (B) R1: well perfused according to indocyanine green (ICG)-augmented hyperspectral imaging (2) (after 6 h). (C) R2: well perfused according to clinical evaluation (3) (after 6 h). (D) R3: well perfused according to conventional ICG imaging (4) (after 6 h). (E) R4: not perfused (after 6 h). Upper scale bar indicates 1 mm, lower scale bar indicates 500 μm. (F) RGB and StO_2_ visualization with indicated regions. (G) DeePathology-based histopathological evaluation. (H), quantification of area of pre-necrotic changes in histopathological samples. Boxplots show mean and standard deviation. A indicates number of animals. Scale bar equals 5 cm.

In order to evaluate whether the differences in HSI oxygenation have any biological relevance and reflect histopathological changes, small bowel tissue was sampled after 6 hours from regions 1–4 (R1–4) and the control region (R control) (Fig. 5). R control was a physiological region. R1 was well perfused according to IA-HSI (2). R2 was well perfused according to clinical evaluation (3). R3 was well perfused according to conventional ICG imaging (4). R4 was not perfused according to any modality. All tissue samples were fixated in 5% formaldehyde (Otto Fischar GmbH & Co. KG) for 24 h and subsequently transferred to 70% ethanol. Specimen were embedded in paraffin, sampled in 5 µm sections and stained with Mayer’s Hematoxylin-Eosin (H/E) according to institutional standard. Slides were scanned using widefield microscopy and the amount of mucosal eosinophilic pre-necrotic areas was objectified with the specialized software DeePathology STUDIO (Deepathology Ltd.) (Fig. 5).

### Vascular corrosion casting and scanning electron microscopy

**Figure 6:**
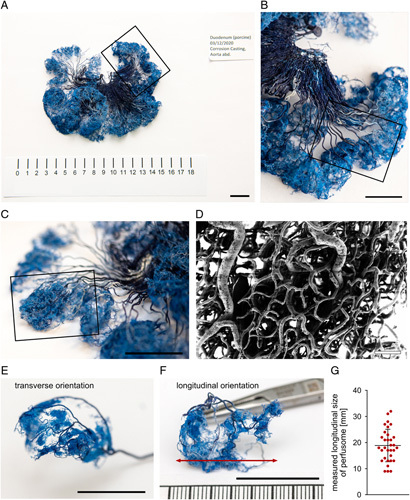
Vascular corrosion casting of small bowel. (A) Small bowel corrosion casting. (B) Subarea of (A). (C) Subarea of (B) indicating the singular perfusome of (E) and (F). (D) Scanning electron microscopy (SEM) recording of perfusome capillaries. (E) Singular perfusome in transverse orientation. (F) Singular perfusome in longitudinal orientation with red arrow indicating measured length. (G) Quantification of measured longitudinal size of perfusomes (A=2, *n*=32). Boxplot shows mean and standard deviation. A indicates number of animals, *n* indicates number of independent measurements. Scale bar in the corrosion casting equals 2 cm. Scale bar in SEM equals 100 μm.

Vascular corrosion casting of the small bowel was obtained using the Biodur E20 kit (Biodur Products) (Fig. 6). It was freshly mixed at a ratio of 100:45 (v/v) of Biodur E20 Plus and catalyst E20. The abdominal aorta was cannulated, the distal thoracic aorta and iliac arteries were clamped and the inferior caval vein was incised after euthanasia with i.v. potassium chloride. While flushing solution (1000 ml Sterofundin ISO by B. Braun/50.000 I.U. Heparin) was injected under mild pressure via the arterial cannulations, blood from the respective circulatory area was collected at the venous outlet. Flushing was continued until significant blood dilution was recognized at the venous outlet. Biodur E20 kit was freshly mixed at a ratio of 100:45 (v/v) of Biodur E20 Plus and catalyst E20 and injected manually. Upon venous return of the casting agent observed in the inferior caval vein, the infusion was stopped and the casting initially hardened for 20 min without further manipulation. The small bowel specimen of the R1 region, as a representation of a physiological small bowel loop, was explanted and incubated for 12 h in a 40°C water bath for further hardening. Excess tissue was removed with 15% (w/v) potassium hydroxide at room temperature for 3 days and the resulting vascular corrosion cast was subsequently rinsed in water. Scanning electron microscopy samples were prepared from small bowel wall. 20×10 mm samples of the outer layer were sputtered with 10 nm gold/platinum (80:20) (Leica EM ACE 600, Leica Microsystems GmbH) and analyzed by scanning electron microscopy (Zeiss Leo Gemini 1530, Carl Zeiss AG). Images were taken at different magnifications with an accelerating voltage of 2.0 kV.

### Informatics and statistical analysis

All the codes were developed and executed with PyCharm 2019.1.2 and Python 3.7. For data organization and storage Microsoft Excel 16.34 has been used. Data visualization and statistical analyses were performed with GraphPad Prism 8.3.1. A *p* value less than 0.05 was conventionally considered statistically significant. Statistical testing was done with multiple comparison testing using ordinary one-way ANOVA for unpaired parametrically distributed data and mixed-effects analysis with Geisser-Greenhouse correction for paired parametrically distributed data with assumed equal standard deviations. Kruskal–Wallis test was used for unpaired non-parametrically distributed data, while Friedman test was used for paired non-parametrically distributed data. Numerical values are provided with standard deviation in brackets. Significance levels were adjusted for multiple testing and were indicated with * for *p* less than or equal to 0.05, ** for p less than or equal to 0.01, *** for p≤0.001, **** for p less than or equal to 0.0001 and n.s. for “not significant”. In the dot plots one dot represents the measurement of one region of interest within one recording if not mentioned otherwise. There was no previous protocol registration.

## Results

### Small bowel baseline measurements

Physiological small bowel (PSB), avascular small bowel (ASB) and small bowel after i.v. application of ICG (ISB) as the three baseline groups of this study showed spectral differences in reflectance. Consequently, also characteristic differences in the colour-coded index images of the HSI system were present (Fig. 1) (Supplement Figure 2, Supplemental Digital Content 1, http://links.lww.com/JS9/A972). The most significant changes in the spectrum could be seen in the direct comparison of spectral reflectance (Fig. 1H). While ISB was relatively similar to PSB with the exception of strongly decreased reflectance values at 800 nm (spectral point of maximum absorbance of ICG) and slightly increased reflectance values at 835 nm (spectral point of maximum emission of ICG)^35^, ASB had a significantly different spectral signature with a loss of the double-peak at 550–600 nm and an emphasized drop at 650 and 750 nm.

Distinct changes in the colour-coded index images of the HSI system were explicitly pronounced for StO_2_ and NIR. Here, the PSB group had values of 67% (±11%) for StO_2_ and 57% (±12%) for NIR, while the ASB group had 19% (±4%) and 8% (±6%) and the ISB group had 80% (±6%) and 45% (±14%) respectively (Supplement Table 1, Supplemental Digital Content 2, http://links.lww.com/JS9/A973).

### Spectral changes during reversible malperfusion

During mesotomy-induced (upper bowel loop; reference loop) as well as during clamp-induced (lower bowel loop) segmental impairment of small bowel perfusion, StO_2_ values dropped as low as 21% (±3%) and 22% (±2%). This shows that the clamp-induced model is an adequate representation of malperfusion through mesotomy. After release of the clamp in the reversible loop, values reproducibly went back up to baseline after 20 min of reperfusion (Fig. 2). At ~2 min after reperfusion StO_2_ values reproducibly climbed to 72% (±6%), which was even above the reference values from the physiological baseline measurements (Supplement Table 2, Supplemental Digital Content 2, http://links.lww.com/JS9/A973).

### Spectral changes during ICG application

Consecutively to i.v. ICG application, StO_2_ values rose to a maximum of 78% (±7%) after 3 min before returning back to baseline values of 61% (±7%) over 120 min (Supplement Table 3, Supplemental Digital Content 2, http://links.lww.com/JS9/A973). An initial flare-up could be seen at 30 seconds as indicated with the white arrow in Figure 3A. These pharmacokinetic characteristics are essential to know when planning to investigate phenomena involving ICG.

### Comparison of HSI, IA-HSI, clinical evaluation and ICG imaging

HSI StO_2_ images of mesotomy-induced segmental impairment of small bowel perfusion (1) were capable of visualizing areas of physiological perfusion and areas of clearly impaired perfusion (Fig. 4). However, exact borders where physiological perfusion started to decrease were hard to clearly identify (Fig. 4D). Instead after i.v. ICG application, IA-HSI of the area of mesotomy-induced segmental impairment of small bowel perfusion (2) (Fig. 4E) suggested a sharp-resection line, where StO_2_ values started to clearly decrease when compared to physiological regions. And while a sharp-resection line does not necessarily imply relevance or validity, it clearly is a sign of a homogenous and uniform process with its relevance and validity requiring confirmation through subsequent histopathological evaluation. Clinical evaluation (3) suggested a resection line 23 mm (±3 mm) and conventional ICG imaging (4) even suggested a resection line 49 mm (±8 mm) closer towards the malperfused region (Fig. 4B-C and Supplement Table 4, Supplemental Digital Content 2, http://links.lww.com/JS9/A973). These 3 borders defined the following four regions (Fig. 4A, D, E): R control (physiological perfusion), R1 (well perfused according to IA-HSI (2)), R2 (well perfused according to clinical evaluation (3)), R3 (well perfused according to conventional ICG imaging (4)) and R4 (not perfused). Corresponding StO_2_ values were 76% (±6%) for R1, 53% (±9%) for R2, 26% (±5%) for R3 and 14% (±2%) for R4 (Fig. 4G) (Supplement Table 5, Supplemental Digital Content 2, http://links.lww.com/JS9/A973). Relevant changes in the reflectance spectra could be observed (Fig. 4H). Interannotator-variability for the measured distances and for the StO_2_ values was low (Supplement Figure 5, Supplemental Digital Content 1, http://links.lww.com/JS9/A972, Supplement Tables 6-8, Supplemental Digital Content 2, http://links.lww.com/JS9/A973). Retrospectively, a recommendable StO_2_ cutoff value as the arithmetic middle between mean values of R1 and R3 was determined to be less than 51% for this specific dataset (Fig. 4G). A principal component analysis for all five regions before and after ICG application could provide a first indication that machine learning algorithms could be able to differentiate between these regions and that the clusters correspond to the baseline measurement clusters from Figure 1 (Supplement Figure 3, Supplemental Digital Content 1, http://links.lww.com/JS9/A972).

### Histopathological analysis indicates relevance of findings

Histopathological samples obtained from R1 to R4 and from a physiological control loop revealed a significant increase of the percentage of pre-necrotic surface area from 18% (±12%) for R3 and 33% (±17%) for R4 compared to around 5% for the other regions (Fig. 5) (Supplement Table 9, Supplemental Digital Content 2, http://links.lww.com/JS9/A973). With the knowledge of significantly increased biologically relevant pre-necrotic changes in tissue for R3, the goal now was to identify an StO_2_ cutoff value with high sensitivity and specificity for biologically relevantly malperfused small bowel after i.v. application of ICG.

### Vascular corrosion casting provides pathophysiological explanation

Vascular corrosion casting could show that the perfusion of the small bowel is organized in slightly overlapping perfusomes and that the mean width of one perfusome of small bowel was 19 mm (±6 mm). Interestingly, with these dimensions, the perfusomes identified with vascular corrosion casting were almost exactly double the measured length of 9 mm (±4 mm) (Supplement Table 4, Supplemental Digital Content 2, http://links.lww.com/JS9/A973) between border I (height of last perpendicular capillary inflow) and border II (height of IA-HSI indicating same StO_2_ levels as compared to healthy reference tissue) in Figure 4. Obviously, this makes perfect sense as the perfusion will travel to both sides of the perpendicular capillary inflow and therefore the perfusome could be expected to be double the size of the length between border I and II.

## Discussion

When using IA-HSI for the evaluation of sufficiency of small bowel perfusion, histopathological necrotic surface area of acceptable small bowel borders was comparable to PSB. In contrast, histopathological necrotic surface area of acceptable small bowel borders evaluated with conventional ICG was significantly higher. Therefore, this tissue is of high-risk for demise. Conventional ICG-guided resection consequently might result in underestimation of the necessary amount of small bowel resection which might lead to the necessity of reoperation and additional loss of overall small bowel length.

In general, ICG is used to provide two types of diagnostic evaluations, that is binary Yes-No-evaluations and gradual evaluations. Examples of binary evaluations are fluorescent cholangiography, identification of lymph node drainage or exclusion of complete intestinal ischaemia. These are diagnostic evaluations for which ICG can provide a clear, intuitive and highly valid result with highly relevant clinical implications^36^. Examples of gradual evaluations are usually addressing the quantification of relatively decreased perfusion levels for example in gastrointestinal organs. For these cases ICG could not convince in the past, mainly due to a lack of standardized application as well as a lack of standardized evaluation without objectively quantifiably reference values^21,37^. For example there are no ICG intensity values known below which the gastrointestinal organ wall will perish over time. Over the last few years there have been some animal studies using advanced imaging technologies to quantify ICG using algorithms and machine learning instead of a simple visualization of ICG. One promising example is the discrimination between arterial and venous bowel ischaemia by time-to-peak analysis using the pharmacokinetic properties of ICG^38^, another example is using maximum fluorescence intensity measurement^39^. However, these have not found their way into clinical routine yet. Also, our results are implying that ICG alone for determining the resection borders is less reliable than clinical evaluation. Although there are publications comparing clinical and ICG-based evaluation of SBM^40^, these typically describe a non-inferiority of ICG. A possible explanation is that in a surgical trial resection might be either guided by clinical or ICG-based evaluation, but any surgeon will include a bowel resectional safety margin of a few millimetres or even centimeters. This might then bypass the diagnostic differences in reliability pointed out in this study.

There have been other animal studies investigating bowel malperfusion using HSI^41^. Barberio *et al*.^42^ could show that HSI yielded more accurate results than fluorescence angiography for the estimation of local lactate values and reported similar HSI StO_2_ values of below 30%. Another study developed a possibility for superposition of the hyperspectral information onto real-time images^43^. Yet, these applications await translation into human practice. While it has been proposed to combine fluorophores with multispectral imaging^44^, to our knowledge, this is the first study to suggest a possible combination of ICG and HSI in that ICG becomes the contrast agent in IA-HSI.

This ICG application causes visual artifacts in the StO_2_ values of the HSI camera such that values significantly increase. This does certainly not resemble a true increase in oxygenation levels and is exclusively caused by the optical properties of systemic ICG. However, this empirical finding can be used to visualize segmental SBM utilizing ICG as contrast agent in an approach for ICG-augmented hyperspectral imaging. Therefore, the most striking limitation of this study is the fact that the HSI system is obviously not used as intended when adding ICG as a contrast agent “off-label”. In fact, the computation of physiological parameters (e.g. the StO_2_ index values) depends on certain assumptions, in particular the presence and absence of chromophores that affect the spectral measurements. More specifically, by introducing exogenous contrast with the injection of ICG, the computations of index values become invalid and physiologically implausible. While we are well-aware of this fact, our work is based on the empirical observation made at the beginning of the pilot experiments that ICG visualizes small bowel loops with physiological perfusion in darker red values on the StO_2_ scale.

A second limitation is the number of 10 pigs for in the main experimental group, which is sufficient considering the large-animal model and the amount of data obtained per animal, but still quite small by measures for clinical relevance. Consistency of the results will have to be evaluated in consecutive studies. Further limitations include that this study was only an animal study and human anatomy might present differently, although small bowel physiology and anatomy is known to be highly comparable.

Furthermore, StO_2_ values and underlying tissue spectra progress as a function of time since ICG administration as shown in Fig. 3. The assessment of the boundary between perfused and non-perfused tissue may thus be subject to temporal fluctuations if the timing between ICG admission and imaging is not standardized. In addition to these temporal fluctuations, other parameters such as body weight, kidney function and ICG dosage may add to the variability of the StO_2_ signal upon ICG administration. The recommended resection borders according to each modality for Figure 4 were placed purely subjectively or even intuitively as described in the methods section and thus, the comparison of such set borders is susceptible to subjective error. As analyzed in other works^45,46^, the placement of such resection lines varies noticeably among practitioners. Therefore, not the exact distance in millimetres is the key message of this study, but the general tendency and implications that can be derived from this. It is also true that the visual inspection with the eye of an experienced surgeon yielded resection borders (III) with necrosis rare comparable to physiological regions. However, the justification for the additional effort that comes with AI-HSI is the objectivity of the method required in a variety of cases such as in minimally invasive surgery with limited visual angles, for guidance of surgeons in training, for prospective data collections during multicenter trials to create long-term evidence as well as for the use in future applications such as artificial-intelligence-based decision-support systems.

Although the determination of the recommended StO_2_ cutoff value at 51% achieves great sensitivity and specificity for this specific dataset, this only holds in this very local context due to a relatively small sample size and its exclusively retrospective fitting, resulting in limited external validity. In order to determine a more globally applicable cutoff value, an in-depth prospective analysis with a hold-out test datasets should be performed in future work.

Certain strengths of this study are a relatively large number of images and the consistent analysis and validation with advanced HSI imaging modalities, histopathology and vascular corrosion casting.

In order to further explore the hypotheses made in this study, one could argue to prospectively test resection margins evaluated by different imaging modalities, create anastomoses and check for insufficiency during a planned relook operation. However, due to the relatively small spatial effects, the continuous nature of a gradual malperfusion and the great number of confounders, this would mean unreasonably high sample sizes that cannot be justified for an exploratory animal study.

While ICG fluorescence is available in many centres, HSI technology is not. Although a direct translation from pigs to patients is not guaranteeable, a possible translation of the results described in this study into clinical practice would include the surgical awareness of the specific deficiencies of conventional ICG. In cases of injuries to the small bowel meso or mesotomy in a normotensive patient that require small bowel sparing resection, the surgeon should be aware that border II (height of IA-HSI indicating same StO_2_ levels as compared to healthy reference tissue with physiological necrosis rates) and border IV (height of ICG imaging suggesting resection by indicating definitive presence of ICG) were 50 mm (±14 mm) apart (Supplement Table 7, Supplemental Digital Content 2, http://links.lww.com/JS9/A973). Consequently, this is the length of small bowel that should potentially be sacrificed from the distal end of ICG-perfusion.

## Conclusion

When comparing ICG to HSI imaging for the evaluation of segmental SBM, StO_2_ images (1) were capable of visualizing areas of physiological perfusion and areas of clearly impaired perfusion. However, exact borders where physiological perfusion started to decrease could not be clearly identified. Instead, IA-HSI (2) suggested a sharp-resection line where StO_2_ values started to decrease. Clinical evaluation (3) suggested a resection line 23.4 mm (±6.6 mm) and conventional ICG imaging (4) even suggested a resection line 53.2 mm (±12.7 mm) closer towards the malperfused region. Histopathological evaluation revealed that the region that was found to be sufficiently perfused by conventional ICG (4), but not by any other method (R3) had a significant increase in pre-necrotic changes in 27.0% (±8.9%) of surface area, which certainly has at least a biological relevance. A retrospectively fitted StO_2_ cutoff value of 51% after ICG application can be suggested for diagnosing seriously malperfused small bowel regions.

IA-HSI (2) is therefore more sensitive for the visualization of segmental SBM compared to conventional HSI imaging (1) or conventional ICG imaging (4) regarding histopathological safety of perfusion and more objective compared to clinical evaluation (3). While the IA-HSI approach might not appear to be the most bowel-length sparing approach, which might be essential in cases of imminent short-bowel syndrome or Crohn’s disease, on a second thought it actually might be due to the possible avoidance of reoperation. Clinical applicability and relevance will have to be explored in future clinical trials.

## Ethical approval

The research related to animal use complies with all the relevant national regulations, institutional policies and has been approved by the authors' institutional review board or equivalent committee in Karlsruhe (G-161/18 and G-262/19).

## Consent

Since this is an exploratory animal study, there was no consent.

## Sources of funding

There was no special funding for this project. Funding from the Willy Robert Pitzer Foundation (grant number: not applicable), the Heidelberg Foundation of Surgery (grant number: not applicable) and the European Research Council (ERC) under the European Union’s Horizon 2020 research and innovation program (NEURAL SPICING, Grant agreement No. 101002198) contributed to this work. Animal experiment approval: The research related to animal use complies with all the relevant national regulations, institutional policies and has been approved by the authors’ institutional review board or equivalent committee in Karlsruhe (G-161/18 and G-262/19). Published data will be made available upon reasonable request.

## Author contribution

A.S.F., F.N. and C.M.H. performed the initial review of existing literature and the planning. A.S.F., C.M.H., K.K., F.M.S. and B.Ö. performed the surgeries. A.S.F., J.O., M.R., L.A., S.S., J.S. and B.Ö. developed the Python code for analysis. A.S.F., F.M.S. M.R., S.S., J.S., L.A., S.K., K.F.K., M.D., G.A.S., H.G.K. and B.Ö. analyzed and interpreted data. F.N., L.M.H. and B.P. provided expert knowledge throughout the project. A.S.F. and F.N. wrote the manuscript. L.M.H., M.R., S.S., J.S., L.A. and B.P. revised the manuscript. All authors have read and approved the final manuscript.

## Conflicts of interest disclosure

Authors state no conflict of interest. Felix Nickel reports support for courses and travel from Johnson and Johnson, Medtronic, Intuitive Surgical, Cambridge Medical Robotics and KARL STORZ as well as consultancy fees from KARL STORZ.

## Research registration unique identifying number (UIN)

Since this is an exploratory animal study, it has not been registered in a publicly accessible database.

## Guarantor

Felix Nickel.

## Provenance and peer review

It was not invited.

## Data availability statement

Published data will be made available upon reasonable request.

## Supplementary Material

SUPPLEMENTARY MATERIAL
