# Supplementary material for: ICG-augmented hyperspectral imaging for visualization of intestinal perfusion compared to conventional ICG fluorescence imaging: an experimental study
Source: Int J Surg. 2023 Sep 14;109(12):3883–95. doi: 10.1097/JS9.0000000000000706 (PMC10720797; doi:10.1097/JS9.0000000000000706)
Supplement: SUPPLEMENTARY MATERIAL [file js9-109-3883-s001.docx]

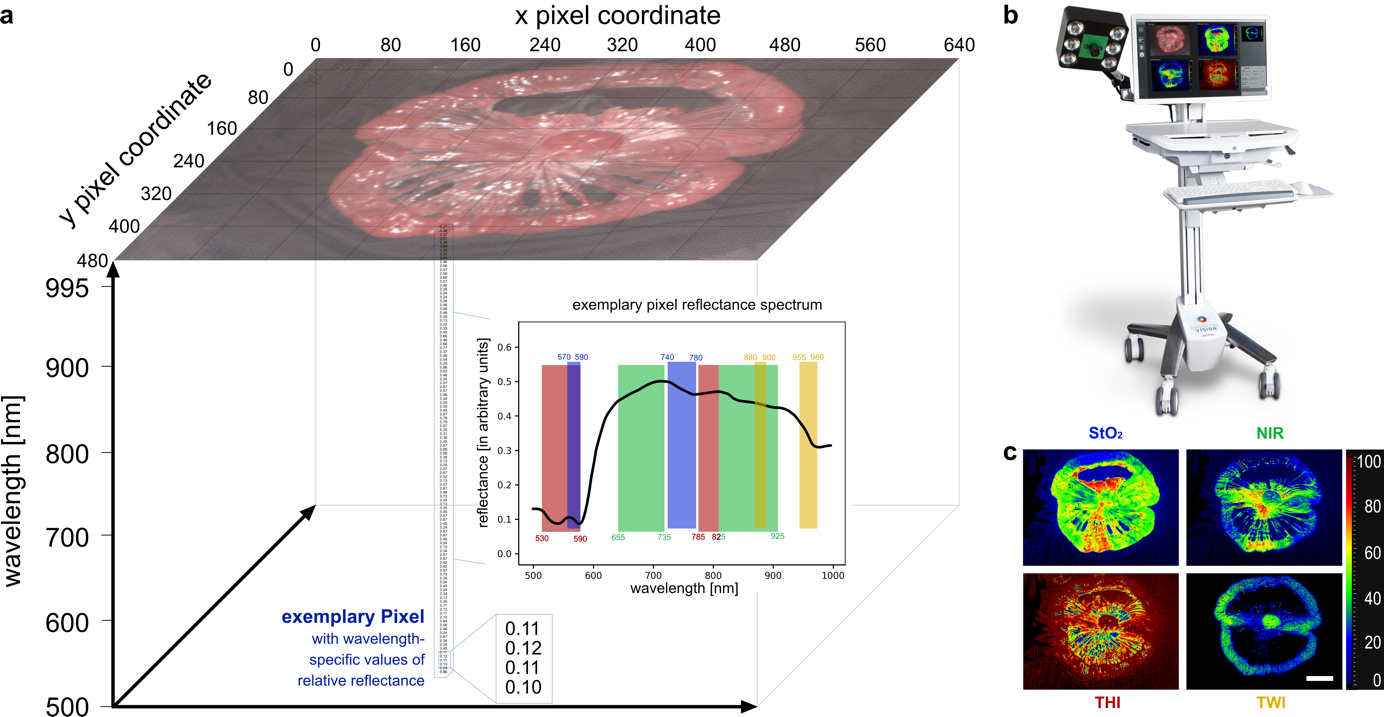


**Supplement Figure 1 | Technology of hyperspectral imaging**. **a**, hyperspectral data structure. **b**, TIVITA Tissue from Diaspective Vision. **c**, StO_2_, NIR, THI and TWI recording. Scale bar equals 5 cm.


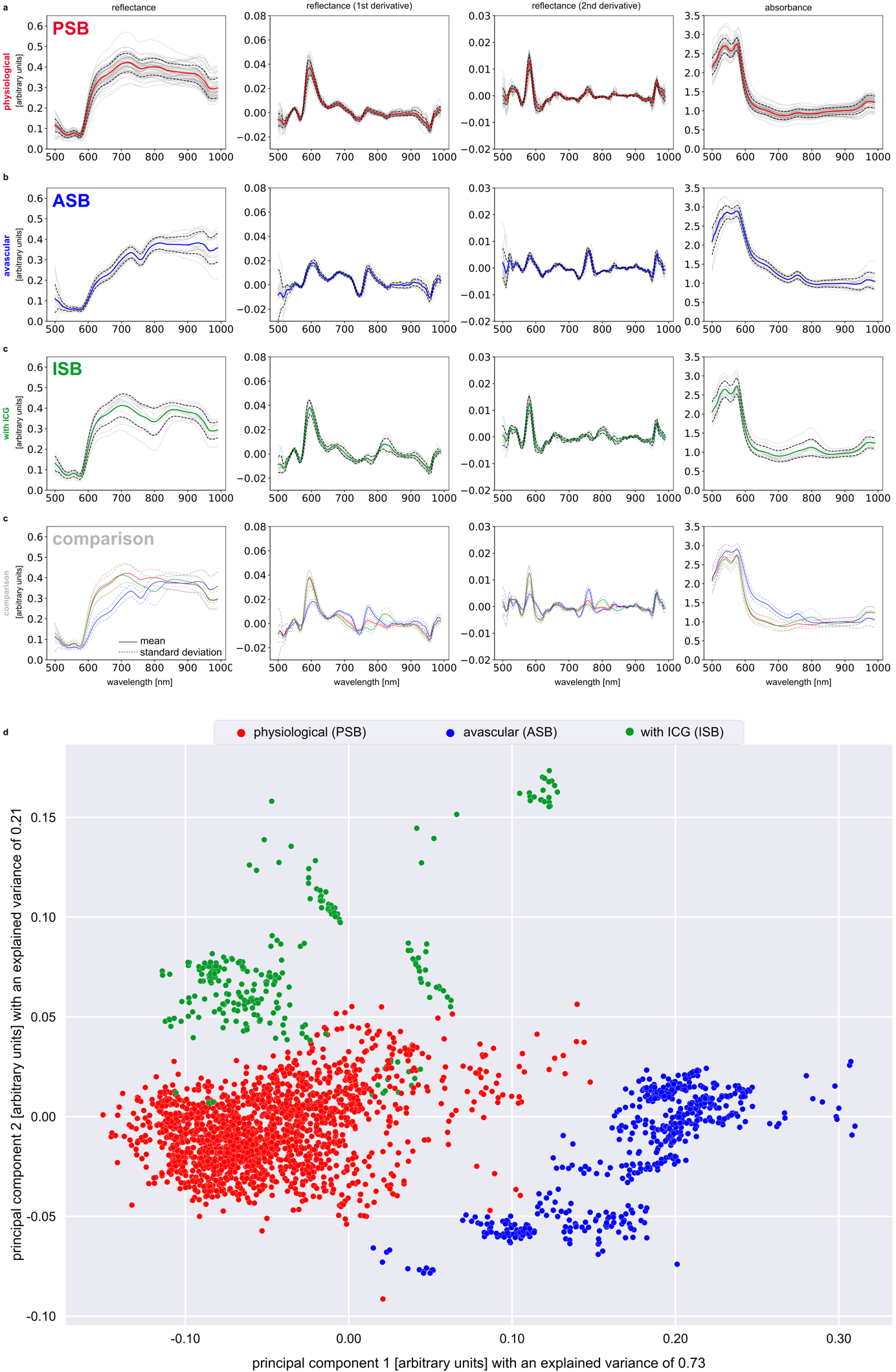


**Supplement Figure 2 | Baseline spectra of small bowel**. Original reflectance, first derivative, second derivative and approximated absorbance. **a**, physiological small bowel (PSB) (A=54, n=1595). **b**, avascular small bowel (ASB) (A=17, n=531). **c**, small bowel with ICG (ISB) (A=13, n =256). **d**, PCA analysis based on baseline small bowel spectra with a total explained variance of 0.94 (0.73 for x; 0.21 for y). A indicates number of animals, n indicates number of independent measurements.


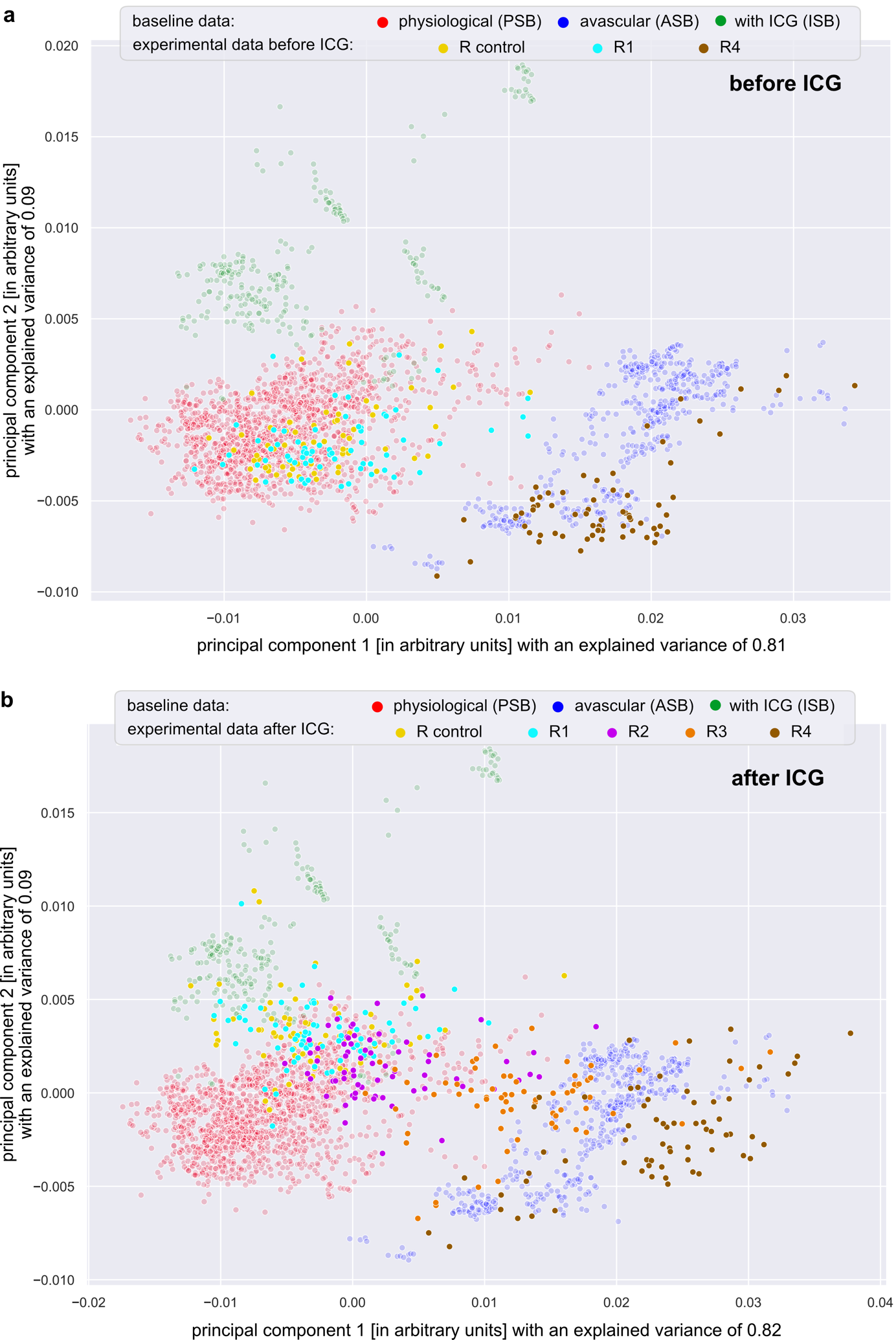


**Supplement Figure 3 | PCA of the 5 experimental regions**. PCA of baseline data in the background. **a**, PCA before ICG with a total explained variance of 0.91 (0.81 for x; 0.09 for y). **b**, PCA after ICG with a total explained variance of 0.91 (0.82 for x; 0.09 for y). Clusters can be clearly differentiated and correspond to the 3 baseline groups (PSB, AVB, ISB).


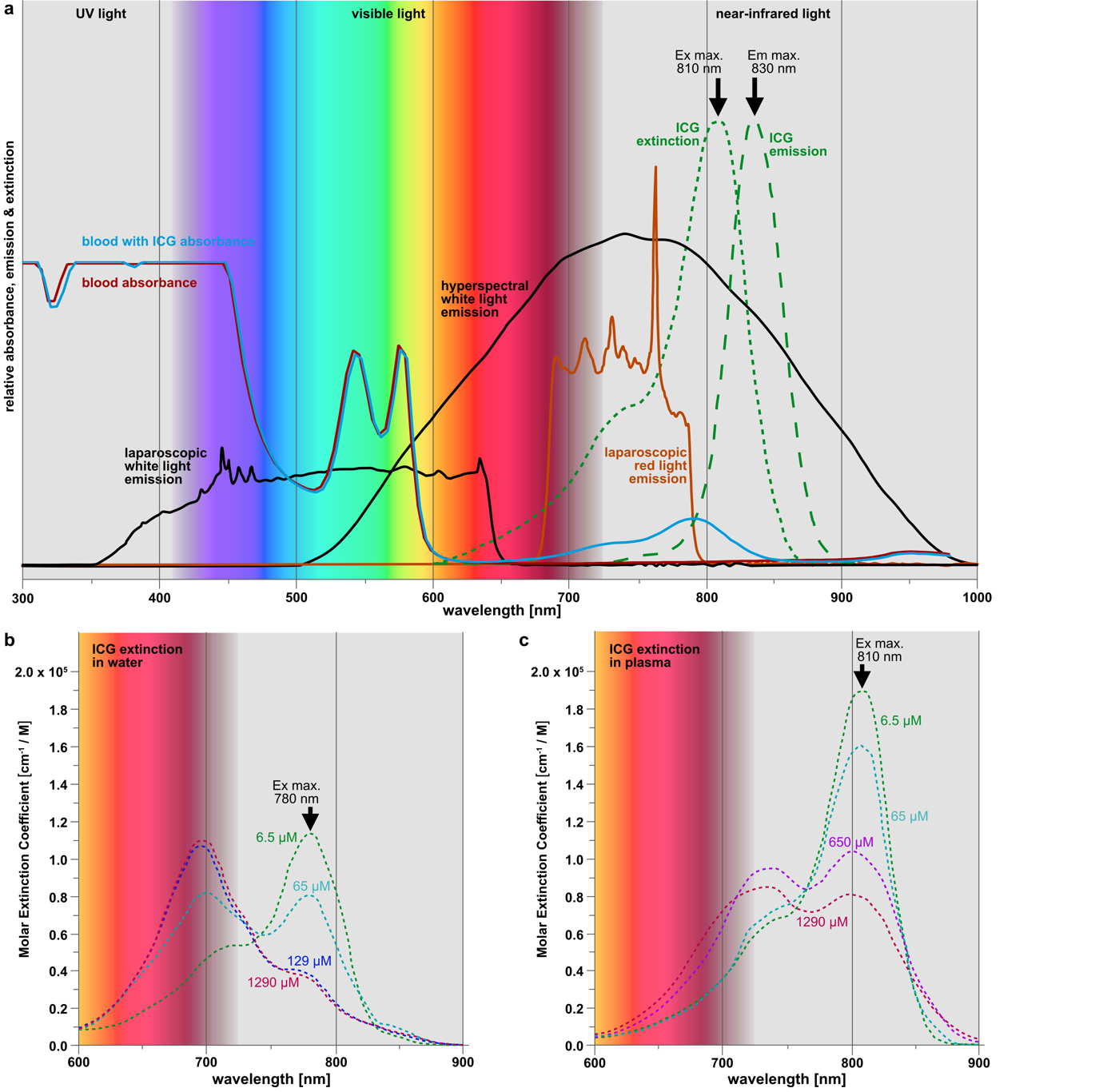


**Supplement Figure 4 | Spectral absorbance, emission and extinction of blood, ICG and light sources. a**, Spectral characteristics of blood (red), blood with ICG (blue), ICG (green), laparoscopic and halogen white light sources (black) as well as laparoscopic ICG light sources (orange). **b**, extinction of ICG in water. **c**, extinction of ICG in blood plasma.


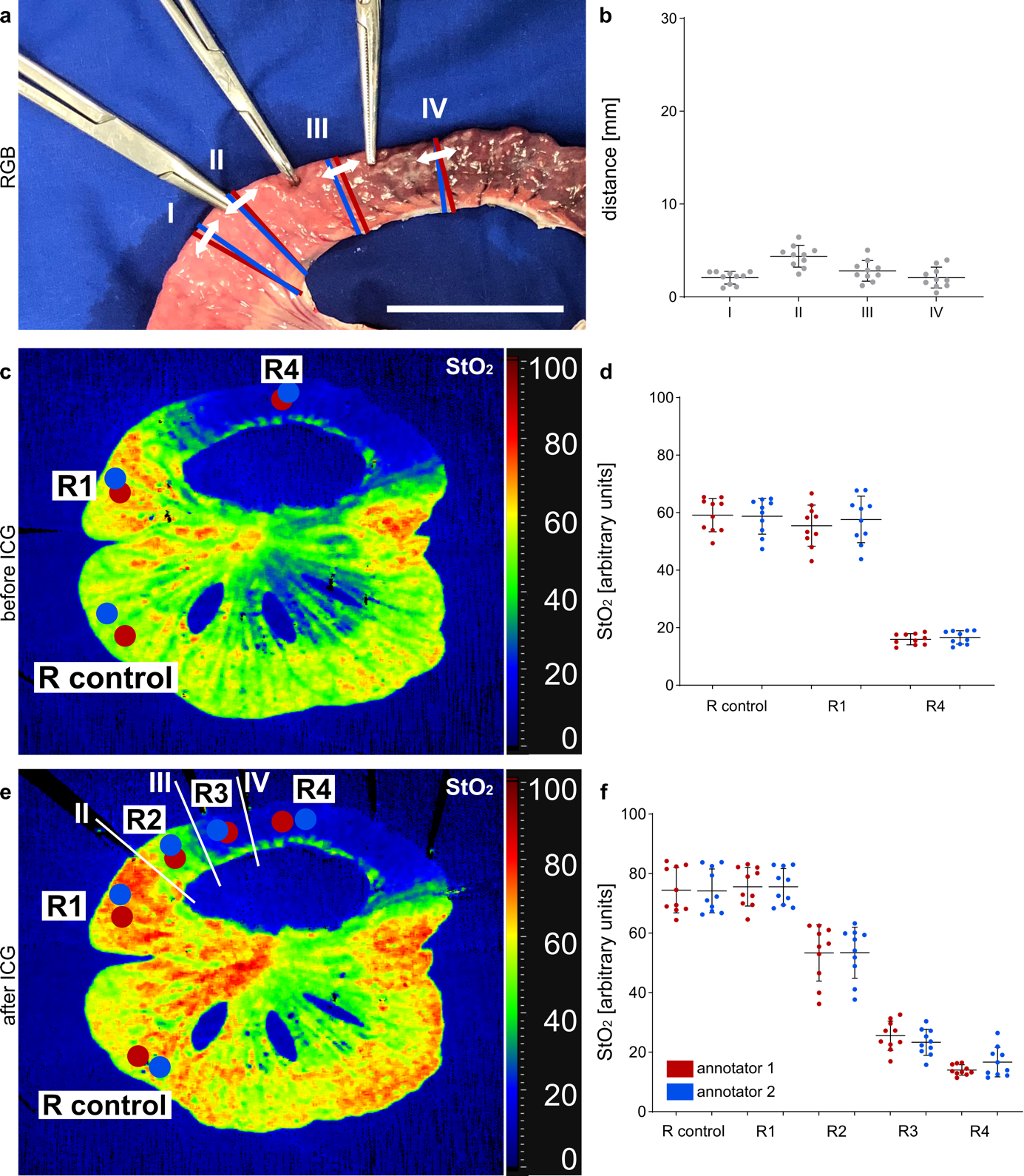


**Supplement Figure 5 | Interannotator-variability of the evaluation of mesotomy-induced segmental impairment of small bowel perfusion.** Annotations were performed by two independent experts (red and blue) and compared. **a**, RGB image with indicated perfusion borders according to different imaging modalities. **b**, quantification of annotator-differences between measured perfusion borders. **c**, visualization before ICG application. **d**, annotator-differences in the StO_2_ quantification before ICG application **e**, visualization after ICG application. **f**, annotator-differences in the StO_2_ quantification after ICG application. Boxplots show mean and standard deviation. Scale bar equals 5 cm.
