# Supplementary material for: ICG-augmented hyperspectral imaging for visualization of intestinal perfusion compared to conventional ICG fluorescence imaging: an experimental study
Source: Int J Surg. 2023 Sep 14;109(12):3883–95. doi: 10.1097/JS9.0000000000000706 (PMC10720797; doi:10.1097/JS9.0000000000000706)
Supplement: SUPPLEMENTARY MATERIAL [file js9-109-3883-s002.docx]

**Supplement**

|  | **physiological** | **avascular** | **with ICG** |
| --- | --- | --- | --- |
| **StO_2_ in %** | 67 (± 11) | 19 (± 4) | 80 (± 6) |
| **NIR in %** | 57 (± 12) | 8 (± 6) | 45 (± 14) |
| **THI in %** | 95 (± 6) | 93 (± 5) | 84 (± 11) |
| **TWI in %** | 55 (± 12) | 26 (± 17) | 63 (± 15) |

**Supplement Table 1 | Baseline of small bowel data**. HSI color-coded index values with mean and standard deviation in percent.

|  | **StO_2_ in %** |
| --- | --- |
| physiological baseline | 54 (± 7) |
| malperfusion 0 min | 45 (± 8) |
| malperfusion 2 min | 38 (± 6) |
| malperfusion 4 min | 32 (± 6) |
| malperfusion 10 min | 26 (± 3) |
| malperfusion 20 min | 22 (± 2) |
| reperfusion baseline | 22 (± 3) |
| reperfusion 0 min | 54 (± 14) |
| reperfusion 2 min | 72 (± 6) |
| reperfusion 4 min | 67 (± 7) |
| reperfusion 10 min | 57 (± 8) |
| reperfusion 20 min | 55 (± 7) |
| reperfusion baseline (of the mesotomy-induced malperfusion loop) | 21 (± 3) |

**Supplement Table 2 | Small bowel malperfusion and reperfusion**. HSI StO_2_ values of the clamp-induced malperfusion loop with mean and standard deviation in percent. HSI StO_2_ values of reperfusion baseline of mesotomy-induced malperfusion loop as an indicator for the equivalence between the clamp-induced and the mesotomy-induced model.

|  | **StO_2_ in %** |
| --- | --- |
| before ICG | 62 (± 8) |
| 0.5 min | 69 (± 12) |
| 1.0 min | 70 (± 13) |
| 1.5 min | 73 (± 10) |
| 2.0 min | 76 (± 8) |
| 3.0 min | 78 (± 7) |
| 5.0 min | 77 (± 6) |
| 15.0 min | 76 (± 5) |
| 30.0 min | 72 (± 8) |
| 45.0 min | 69 (± 7) |
| 60.0 min | 67 (± 7) |
| 90.0 min | 66 (± 6) |
| 120.0 min | 61 (± 7) |

**Supplement Table 3 | ICG progression over time**. HSI StO_2_ values with mean and standard deviation in percent.

|  | **I-II** | **II-III** | **III-IV** |
| --- | --- | --- | --- |
| distance^1^ | 9 mm (± 4 mm) | 23 mm (± 7 mm) | 30 mm (± 11 mm) |
| distance^2^ | 8 mm (± 3 mm) | 23 mm (± 3 mm) | 26 mm (± 7 mm) |
|  | **I-III** | **II-IV** | **I-IV** |
| cumulative distance^1^ | 32 mm (± 7 mm) | 53 mm (± 13 mm) | 62 mm (± 13 mm) |
| cumulative distance^2^ | 31 mm (± 4 mm) | 49 mm (± 8 mm) | 57 mm (± 9 mm) |

**Supplement Table 4 | Spatial evaluation of mesotomy-induced segmental impairment of small bowel perfusion**. Distances between the borders I to IV from annotator 1. ^1^ indicates values from raw data. ^2^ indicates values from hierarchical aggregation.

|  | **R control** | **R1** | **R2** | **R3** | **R4** |
| --- | --- | --- | --- | --- | --- |
| before ICG^1^ | 65 (± 7) | 63 (± 9) |  |  | 17 (± 4) |
| before ICG^2^ | 59 (± 6) | 55 (± 7) |  |  | 16 (± 2) |
| after ICG^1^ | 83 (± 8) | 83 (± 8) | 57 (± 10) | 27 (± 9) | 14 (± 3) |
| after ICG^2^ | 74 (± 8) | 76 (± 6) | 53 (± 9) | 26 (± 5) | 14 (± 2) |

**Supplement Table 5 | Evaluation of oxygenation of mesotomy-induced segmental impairment of small bowel perfusion**. HSI StO_2_ values in % for R control and R1 to R4 with mean and standard deviation in percent from annotator 1. ^1^ indicates values from raw data. ^2^ indicates values from hierarchical aggregation.

|  | **I** | **II** | **III** | **IV** |
| --- | --- | --- | --- | --- |
| distance | 2 mm (± 1 mm) | 4 mm (± 2 mm) | 3 mm (± 2 mm) | 2 mm (± 1 mm) |

**Supplement Table 6 | Inter-annotator distances of evaluated borders**. Difference in distances between the borders I to IV after from hierarchical aggregation.

|  | **I-II** | **II-III** | **III-IV** |
| --- | --- | --- | --- |
| distance^1^ | 11 mm (± 5 mm) | 21 mm (± 6 mm) | 29 mm (± 12 mm) |
| distance^2^ | 11 mm (± 4 mm) | 20 mm (± 3 mm) | 28 mm (± 4 mm) |
|  | **I-III** | **II-IV** | **I-IV** |
| cumulative distance^1^ | 32 mm (± 7 mm) | 50 mm (± 14 mm) | 61 mm (± 13 mm) |
| cumulative distance^2^ | 31 mm (± 4 mm) | 48 mm (± 6 mm) | 59 mm (± 7 mm) |

**Supplement Table 7 | Spatial evaluation of mesotomy-induced segmental impairment of small bowel perfusion**. Distances between the borders I to IV from annotator 2. ^1^ indicates values from raw data. ^2^ indicates values from hierarchical aggregation.

|  | **R control** | **R1** | **R2** | **R3** | **R4** |
| --- | --- | --- | --- | --- | --- |
| before ICG^1^ | 62 (± 9) | 61 (± 9) |  |  | 16 (± 3) |
| before ICG^2^ | 59 (± 6) | 58 (± 8) |  |  | 17 (± 2) |
| after ICG^1^ | 80 (± 8) | 80 (± 8) | 56 (± 11) | 22 (± 9) | 15 (± 5) |
| after ICG^2^ | 74 (± 7) | 76 (± 6) | 53 (± 9) | 23 (± 4) | 17 (± 5) |

**Supplement Table 8 | Evaluation of oxygenation of mesotomy-induced segmental impairment of small bowel perfusion**. HSI StO_2_ values in % for R control and R1 to R4 with mean and standard deviation in percent from annotator 2. ^1^ indicates values from raw data. ^2^ indicates values from hierarchical aggregation.

|  | **percentage of pre-necrotic area** |
| --- | --- |
| **R control** | 4% (± 3%) |
| **R1** | 6% (± 4%) |
| **R2** | 6% (± 3%) |
| **R3** | 18% (± 12%) |
| **R4** | 33% (± 17%) |

**Supplement Table 9 | Histopathological correlation of segmental small bowel malperfusion**. Table provides the proportion of microscopic surface area with pre-necrotic changes in percent.
